# Differential Expression of Candidate Salivary Effector Genes in Pea Aphid Biotypes With Distinct Host Plant Specificity

**DOI:** 10.3389/fpls.2019.01301

**Published:** 2019-10-22

**Authors:** Hélène Boulain, Fabrice Legeai, Julie Jaquiéry, Endrick Guy, Stéphanie Morlière, Jean-Christophe Simon, Akiko Sugio

**Affiliations:** ^1^INRA, UMR1349, Institute of Genetics, Environment and Plant Protection, Le Rheu, France; ^2^University of Rennes 1, Inria, CNRS, IRISA, Rennes, France

**Keywords:** host-specificity, transcriptomics, effectors, copy number variation, phytophagous insects

## Abstract

Effector proteins play crucial roles in determining the outcome of various plant-parasite interactions. Aphids inject salivary effector proteins into plants to facilitate phloem feeding, but some proteins might trigger defense responses in certain plants. The pea aphid, *Acyrthosiphon pisum*, forms multiple biotypes, and each biotype is specialized to feed on a small number of closely related legume species. Interestingly, all the previously identified biotypes can feed on *Vicia faba*; hence, it serves as a universal host plant of *A. pisum*. We hypothesized that the salivary effector proteins have a key role in determining the compatibility between specific host species and *A. pisum* biotypes and that each biotype produces saliva containing a specific mixture of effector proteins due to differential expression of encoding genes. As the first step to address these hypotheses, we conducted two sets of RNA-seq experiments. RNA-seq analysis of dissected salivary glands (SGs) from reference alfalfa- and pea-specialized *A. pisum* lines revealed common and line-specific repertoires of candidate salivary effector genes. Based on the results, we created an extended catalogue of *A. pisum* salivary effector candidates. Next, we used aphid head samples, which contain SGs, to examine biotype-specific expression patterns of candidate salivary genes. RNA-seq analysis of head samples of alfalfa- and pea-specialized biotypes, each represented by three genetically distinct aphid lines reared on either a universal or specific host plant, showed that a majority of the candidate salivary effector genes was expressed in both biotypes at a similar level. Nonetheless, we identified small sets of genes that were differentially regulated in a biotype-specific manner. Little host plant effect (universal vs. specific) was observed on the expression of candidate salivary genes. Analysis of previously obtained genome re-sequenced data of the two biotypes revealed the copy number variations that might explain the differential expression of some candidate salivary genes. In addition, at least four candidate effector genes that were present in the alfalfa biotype but might not be encoded in the pea biotype were identified. This work sets the stage for future functional characterization of candidate genes potentially involved in the determination of plant specificity of pea aphid biotypes.

## Introduction

A large majority of herbivorous insects feeds on specific host plant species (Forister et al., 2015). Host plants not only provide food sources but may also provide insect habitat and mating sites. Such continuous and intimate interactions with certain plant species are considered as major driving forces in insect evolution and specialization to host plants, potentially leading to new species through reduction of gene flow between plant-specialized populations and mechanisms reinforcing reproductive isolation (Butlin and Smadja, 2017). Understanding the adaptation mechanisms of insects to their host plants is of paramount importance to increase knowledge on the role of natural selection in species formation but also to contribute to applied issues, notably to respond to the increasing need to develop sustainable crop pest-management strategies. However, the molecular mechanisms of insect specialization to host plant species are little understood, and these mechanisms seem to vary between combinations of plant and insect species (Simon et al., 2015; Birnbaum and Abbot, 2018).

Aphids are major crop pests worldwide and have a very specialized feeding style. Most aphid species have a narrow range of host plants (Peccoud et al., 2010). Aphids feed on plant sap by using their needle-like mouthparts, called stylets. In the process of inserting the stylets into phloem sieve cells and establishing phloem feeding, aphids puncture various plant cells and secrete watery saliva that contains a battery of proteins, many of them expressed in salivary glands (SGs) (Moreno et al., 2011; Boulain et al., 2018). Several salivary proteins were shown to increase aphid fecundity when expressed in plants or to reduce aphid fecundity when their expression was silenced in aphids, providing evidence that these proteins function like effectors of microbial pathogens (Mutti et al., 2006; Mutti et al., 2008; Bos et al., 2010; Pitino et al., 2011; Atamian et al., 2012; Pitino and Hogenhout, 2013; Elzinga et al., 2014; Naessens et al., 2015; Wang et al., 2015; Kettles and Kaloshian, 2016; Guy et al., 2016). *In planta* expression of the salivary effectors C002, Mp1, and Mp2 from the generalist aphid *Myzus persicae* increases the fecundity of *M. persicae* on its host plants *Arabidopsis thaliana* and *Nicotiana benthamiana*, while expression of orthologous genes from a legume-specialist species (*Acyrthosiphon pisum*) in these plants has no effect on *M. persicae* fecundity, suggesting host-specific functions of some salivary proteins (Pitino and Hogenhout, 2013). On the other hand, *in planta* expression of aphid salivary proteins (e.g., Mp10 and Mp42 from *M. persicae*) reduces aphid fecundity, suggesting a possible property of salivary proteins as avirulence proteins, which are recognized by a plant and trigger plant defense reactions against aphids (Bos et al., 2010). These results indicate that a set of salivary effectors can determine the outcome of plant-aphid interactions.

*Acyrthosiphon pisum* is a model aphid species and is often regarded as a single insect species. However, the *A. pisum* complex actually encompasses at least 15 biotypes with differential fitness on specific host plants (Peccoud et al., 2009; Peccoud et al., 2015). Each biotype is specialized to one or a few legume species and cannot perform well on other plants (Peccoud et al., 2009). They have a similar but distinct genetic makeup; therefore, *A. pisum* biotypes are an ideal system for studying the mechanisms of aphid specialization to host plants. Interestingly, all the 15 biotypes feed well on *Vicia faba*, which is considered as a universal host plant of *A. pisum*. Previous analysis of 390 microsatellite markers (Jaquiéry et al., 2012) and pool-seq analysis (Nouhaud et al., 2018) of three *A. pisum* biotypes represented by 60 individual aphids, both indicated that the genomic regions that are highly differentiated between the biotypes are significantly enriched in candidate salivary effector genes. In addition, gene expression analysis of six biotypes of *A. pisum* showed that a relatively high proportion of candidate salivary effector genes is differentially expressed (DE) between the biotypes (Eyres et al., 2016). These studies indicate potential involvement of the salivary effector genes in host plant specialization.

Previously, we conducted transcriptomics analysis and bioinformatics prediction of secreted proteins of the *A. pisum* reference line LSR1 (alfalfa biotype) and identified 3,603 SG-expressed candidate salivary effector genes (Boulain et al., 2018), of which, 740 were upregulated in the SGs compared to the alimentary tract (AT). Proteomics analysis of aphid-fed diet also identified 51 secreted proteins, all of them expressed in the SGs. A comparative genomic analysis using 17 arthropod genomes revealed that the SG-upregulated effector set contains a high proportion of aphid lineage-specific genes and tends to evolve faster. The study also revealed that the salivary effector set was enriched with members of gene families, some of which were expanded in the pea aphid genome compared to other aphid species (Boulain et al., 2018).

Based on the accumulated results of functional characterization of aphid salivary effector proteins and genome-wide analyses of *A. pisum*, we hypothesized that *A. pisum* biotypes express different salivary effector proteins and that biotype-specific mixture of salivary proteins might be required for host plant adaptation. To characterize biotype-specific differences in salivary effector composition and expression level, we conducted transcriptomic analysis of two *A. pisum* biotypes on both the universal (*V. faba*) and specific host plants.

We have chosen the pea biotype to compare with the alfalfa biotype, which includes the reference line LSR1, because they are closely related (limiting the chances to identify highly differentiated genes that are not involved in host specificity) (Peccoud et al., 2009), show distinct phenotypes on the two specific host plants, and various genetic resources and techniques are available in pea (*Pisum sativum*), which will facilitate the follow-up study of effector functions (Guy et al., 2016; Meziadi et al., 2016; Meziadi et al., 2017). As a first step to compare these two biotypes, we created a list of salivary genes using an *A. pisum* pea-adapted line because the previous candidate salivary gene list was created only for the alfalfa-adapted line, LSR1 (Boulain et al., 2018), and may not include the salivary genes that are specific to the pea biotype. To take into account aphid lineage-specific expression differences and to identify the genes that show biotype-specific differential expression patterns, we conducted a transcriptomic study using three genetically distinct aphid lines for each biotype. We also examined the effect of feeding plants (universal host *V. faba* vs. specific host) on the expression patterns of identified salivary genes. Due to the enormous task of dissecting SGs to provide a sufficient amount of RNA for RNA-seq, we used aphid head samples to examine the transcriptome of three aphid lines *per* biotype and the effect of host plants. Nonetheless, we were able to successfully identify salivary genes that are DE in a biotype-specific manner and evaluate the impact of host plants on the expression pattern of salivary genes.

## Materials and Methods

### Aphids, Plants, and Growth Conditions

To explore biotype effects, we studied six different lines of *A. pisum*, of which three lines represented each biotype (**Supplementary Table S1**). To avoid the potential influence of secondary symbionts on overall aphid fitness and plant exploitation mechanisms, we used aphid lines that were free of facultative symbiont. The six aphid lines used in this study, including the LSR1 line for which the genome is sequenced (The International Aphid Genomics Consortium, 2010) were maintained in a growth chamber at 18°C with a 16-h-day/8-h-night photoperiod on their universal host, the broad bean, *V. faba* (cv. Castel), at low density to avoid the production of winged individuals. All plants were grown in a growth chamber at 18°C with a 16-h-day/8-h-night photoperiod. Before installing the aphids for the experiments, *V. faba* and pea, *P. sativum* (cv. Baccara), were grown for 10 days whereas alfalfa, *Medicago sativa* (cv. Comète), was grown for 4 weeks.

### Aphid Performance Assays

Adult aphids from the six lines were installed on each tested plant (*V. faba*, *P. sativum, M. sativa*) so that the nymphs produced did not experience a switch of host plant species. One 1-day-old aphid nymph was installed on each test plant (with 12 test plants *per* condition), and their offspring were counted 18 days later. The experiment was conducted in a growth chamber at 18°C with a 16-h-day/8-h-night photoperiod.

Differences in numbers of offspring produced by each aphid line on the three tested plants were analyzed with a Kruskal-Wallis test performed in R (R Development Core Team, 2017).

### RNA Sequencing

To prepare RNA samples from SGs and ATs of the pea biotype, we used 9-day-old aphids from the line P123 reared at a low density of 10–15 aphids *perV. faba* plant. The aphids were dissected in saline solution. Dissected organs were soaked in RNA later (QIAGEN) to avoid RNA degradation and pooled in batches before RNA extraction (three replicates *per* line and *per* organ). On average, RNA samples from 200 pairs of SGs or 20 ATs that were dissected on the same day were pooled for one replicate of an RNA-seq experiment. Three biological replicates *per* condition were prepared.

To prepare RNA samples from heads of the three alfalfa biotype lines (LSR1, LL01, L84) and the three pea biotype lines (ArPo58, P123, S1PS02), we used 9-day-old aphids reared since birth on the universal host *V. faba* and on the specific hosts (*P. sativum* or *M. sativa*) at a density of 10 aphids *per* plant. Aphids were then collected, flash frozen in liquid nitrogen and heads (in front of the first pair of legs) were cut by scalpel while whole aphid bodies were frozen. Three replicates *per* line and *per* plant were prepared. On average, 20 aphid heads harvested on the same day were pooled for one replicate. Three replicates *per* condition were prepared.

RNA from SGs, ATs, and heads were extracted by NucleoSpin RNA XS (Macherey-Nagel) and quantified. rRNA depletion, single stranded-RNA library preparation, multiplexing, and sequencing were performed by Genewiz (New Jersey, USA). Sequencing was performed on the Illumina HiSeq2500 platform, with a 2 × 125 bp paired-end (PE) configuration in the High Output mode (V4 chemistry). Each sample was sequenced on four different flowcell lanes to avoid lane effect. In total, 269,440,904 reads were obtained for the six SG samples, 257,744,832 reads for the three AT samples, and 1,678,378,894 reads for the 33 head samples. Raw data are available in NCBI Sequence Read Archive (https://trace.ncbi.nlm.nih.gov/Traces/sra/) with reference numbers shown in **Supplementary Table S2**.

### *De Novo* Assembly

Reads from the three SG samples from P123 (this study) and LSR1 (Boulain et al., 2018) were trimmed using trimmomatic (version 0.36, options ILLUMINACLIP:TruSeq3-PE-2.fa:2:30:10 LEADING:3 TRAILING:3 SLIDINGWINDOW:4:15 MINLEN:36), and an assembly for each biotype was done using Trinity (v2.5.1) (Grabherr et al., 2011). Lowly expressed contigs were removed by applying a filter with RSEM (--fpkm_cutoff 0.5, –isopct_cutoff=15.0) (Li and Dewey, 2011). The remaining contigs were mapped on the LSR1 reference genome (The International Aphid Genomics Consortium, 2010) with gmap (version 2018-03-25) (Wu et al., 2016).

Unmapped contigs from each LSR1 and P123 SG library were searched against the nonredundant protein database using a blastx (BLAST+ v2.5.0, e-value = 1e-8) (Camacho et al., 2009) and P123 contigs were blasted against LSR1 contigs to identify those unmapped contigs that were similar between both aphid lines (blastn, e-value = 1e-8).

### Read Mapping and Gene Expression Analysis

The gene expression patterns of *A. pisum* SG, AT, and head samples were analyzed using the Acyr_2.0 reference genome assembly (GCF_000142985.2) with the NCBI *A. pisum* Annotation Release 102, both available at ftp://ftp.ncbi.nlm.nih.gov/genomes. The PE reads were mapped on the reference genome using STAR v2.5.2 (Dobin et al., 2013) with the following parameters: outFilterMultimapNmax=5, outFilterMismatchNmax=3, alignIntronMin=10, alignIntronMax=50,000, alignMatesGapMax=50,000. Subread featureCounts (Liao et al., 2014) was used to estimate fragment counts *per* gene using default parameters. Because some viruses might be associated with adaptation of the pea aphid to its host plants (Lu et al., 2019), reads were also mapped to the genomes of the eight known aphid viruses: the *Acyrthosiphon pisum* virus (NC_003780.1), the *Rhopalosiphum padi* virus (NC_001874.1), the *Brevicoryne brassicae* virus (NC_009530.1), the rosy apple aphid virus (DQ286292.1), the *Aphis glycines* virus 2 (NC_028381.1), the *Macrosiphum euphorbiae* virus 1 (NC_028137.1), the *Myzus persicae* densovirus (NC_005040.1), and the *Dysaphis plantaginea* densovirus (NC_034532.1).

Three gene expression analyses were conducted separately (with SGs and ATs only, with LSR1 and P123 heads only and finally with all heads) following previously described workflows (Chen et al., 2016; Lun et al., 2016). First, the raw fragment counts were converted to counts *per* million (CPM) using the edgeR (Robinson et al., 2010) R-implemented package (R Development Core Team, 2017). Expressed genes were filtered based on a CPM > 1 in at least three of the libraries incorporated in the analysis and then CPMs were normalized using the edgeR TMM method for Normalization Factor calculation (Robinson and Oshlack, 2010). The reproducibility of replicates was then assessed by multidimensional scaling (MDS) of distances between gene expression profiles based on filtered and normalized CPMs (Ritchie et al., 2015). Filtering, normalization, and clustering steps realized for the different analyses are presented in **Supplementary Figures S1**, **S2**, and **S3**. The MDS analysis revealed three head samples (two replicates of P123 and one replicate of S1PS02 both from pea plant condition) that did not cluster with other pea biotype samples. These three samples were removed before further analyses (**Supplementary Figure S3**, **Supplemental Table S2**). Based on the different analyses, we defined a set of 13,203 *A. pisum* genes that were expressed in at least one condition (CPM > 1) and considered as our working gene set.

### Differential Expression Analyses

The differential expression between samples was then explored with different functions implemented in edgeR that allowed us to i) estimate the common dispersion among the data, ii) fit a quasi-likelihood negative binomial generalized log-linear model to the data, and iii) perform empirical Bayes quasi-likelihood F-tests to determine DE genes (Lund et al., 2012). Statistical tests were taken into account only when expression level averages were above CPM >1 in at least one of the conditions that were compared; otherwise, comparisons were treated as nonsignificant. Fold changes (FCs) between conditions were calculated from average CPM and a FC threshold was fixed at 1.5 to be considered as a DE gene. P-values of the statistical tests were adjusted using the False Discovery Rate (FDR) (Benjamini and Hochberg, 1995). A first contrast matrix was designed to test for organ effect (SGs vs. ATs) in LSR1 and P123 lineages and therefore identify the genes that show upregulated expression in SGs compared to ATs. Then, contrast matrices were designed to analyze the plant (universal vs. specific), biotype (pea vs. alfalfa), and line effects among the head samples of the six *A. pisum* lines. The plant and line effects were tested within each biotype whereas the biotype effect was tested between biotypes. DE genes were retained based on the FC and FDR from edgeR as previously described, except for testing the biotype effect. As we noticed that some genes showing intra-biotype variability were still present in our biotype-DE set of genes, we applied a Student t-test after the edgeR statistical test (calculated on average CPM from each line) and filtered the DE genes based on a p-value < 0.05 in both methods.

### Secretion Prediction and Orthology Analysis

Signal peptides and nonclassical secretion signals of *A. pisum* proteins were identified using a combination of SignalP v3.0, v4.1 (Bendtsen et al., 2004b; Petersen et al., 2011) and SecretomeP v2.0 (Bendtsen et al., 2004a), as described by Boulain et al. (2018). Then, among these proteins that are predicted to be secreted, the ones containing membrane-inserted domains such as transmembrane domains (Krogh et al., 2001) or GPI anchors (Pierleoni et al., 2008) were removed as they are likely not secreted.

To assign an orthology level to each *A. pisum* gene, we determined groups of orthologs among 17 arthropod genomes (Boulain et al., 2018). The longest protein isoforms from each arthropod species were used to run OrthoDB_soft_1.6 (Kriventseva et al., 2015) and the levels of orthology were assigned referring to the species phylogeny established by Boulain et al. (2018). The differences in orthologous categories between salivary effector subsets and other genes were then analyzed using proportion tests implemented in R. The groups of orthologs generated by OrthoDB were also used to identify *A. pisum* unique (single copy) or duplicated (multiple copy) genes. Then, we examined whether the salivary effector sets contained more duplicated genes than expected by chance alone. A significant effect was demonstrated if the number of genes that were duplicated lay above the 95% confidence interval (CI) of the expectation. In addition, 95% CI was computed by randomly sampling the number of genes contained in each salivary effector subset (152, 103, and 3,291 for alfalfa-up, pea-up, and non-DE, respectively) from the list of 3,546 salivary effector genes and counting the number of duplicated genes in this random sample. This step was repeated 10,000 times.

### Copy Number Variation Analysis

Population genomic data from Nouhaud et al. (2018), that consisted of Illumina sequencing of two pools of 60 pea- or alfalfa-adapted genotypes (pool-seq) with coverage values >110X each, were used to evaluate copy number variation. The reads from pea and alfalfa biotype pools were mapped following the protocol described by Nouhaud et al. (2018), only primary alignments were kept, and low-quality mapping (q < 20) and identically located reads resulting from PCR duplication were removed with MarkDuplicates from Picard tools (http://broadinstitute.github.io/picard/). A mean coverage for each exon for each biotype was then computed with Bedtools coverageBed (Quinlan, 2014) with the mean option. The mean coverage of each gene was then calculated by summing its exon coverages and dividing by its total exon size. Then, for the purpose of normalization, these coverages were divided by the average coverage of each gene calculated separately on each biotype pool. Finally, the ratio of coverages was computed for each gene using the normalized mean coverages obtained on both pools.

## Results

### Plant Specialization of *A. Pisum* Lines That Belong to Alfalfa and Pea Biotypes

**Figure 1:**
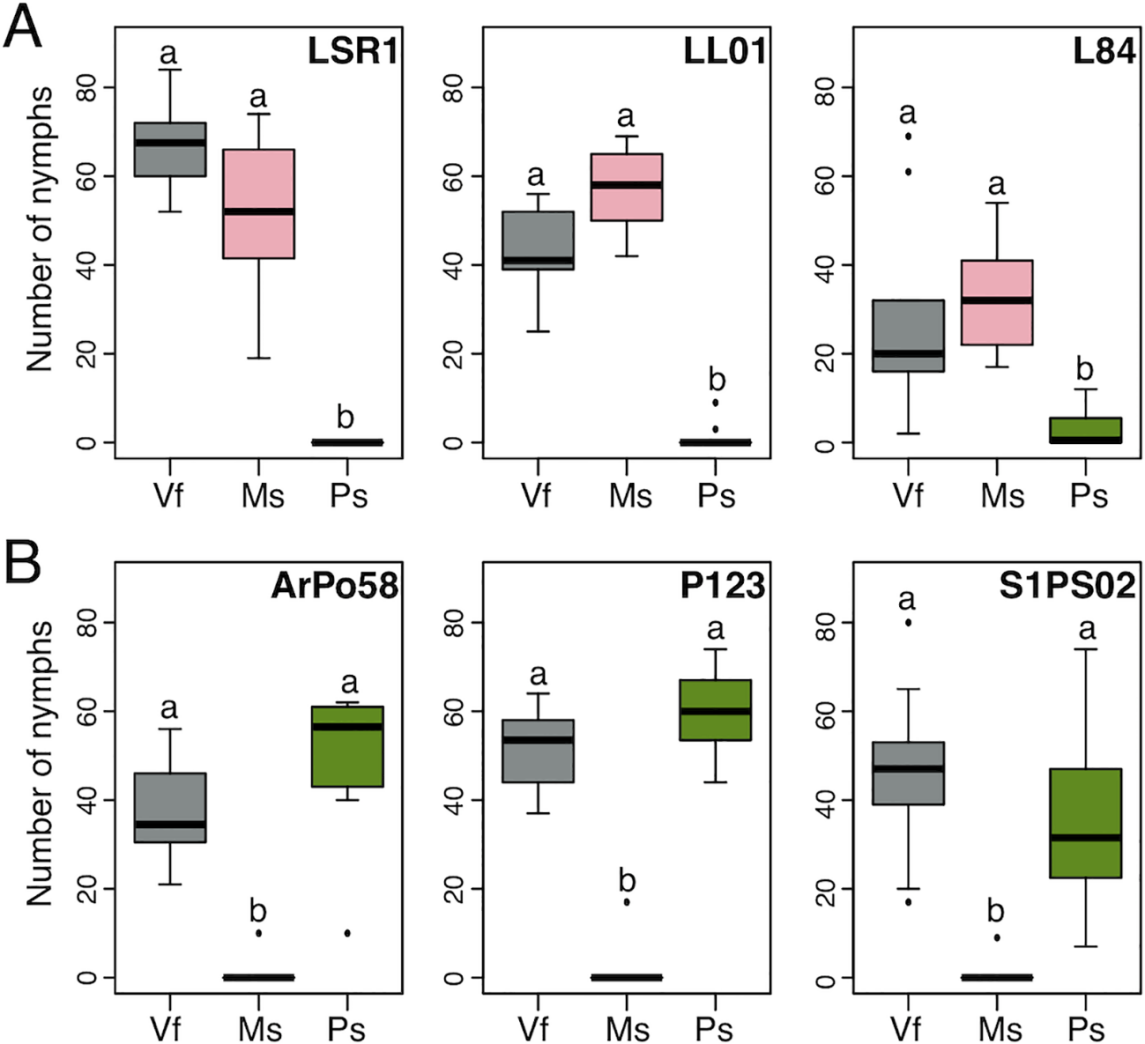
*Acyrthosiphon pisum* lines that belong to the alfalfa **(A)** or pea **(B)** biotype show distinct host specificity. The numbers of nymphs produced by single aphid mother during 18 days on the universal host plant *Vicia faba* (Vf), specific host plants *Medicago sativa* (Ms), and *Pisum sativum* (Ps) are shown. Letters above boxes indicate significant differences determined by multiple Kruskal-Wallis tests for each aphid line (in LSR1: *H* = 25.235, 2 d.f., *P* < 0.001; in LL01: *H* = 26.479, 2 d.f., *P* < 0.001; in L84: *H* = 21.499, 2 d.f., *P* < 0.001, in ArPo58: *H* = 25.778, 2 d.f., *P* < 0.001; in P123: *H* = 23.416, 2 d.f., *P* < 0.001 and in S1PS02: *H* = 23.179, 2 d.f., *P* < 0.001).

We selected three aphid lines (LSR1, LL01, and L84) identified as alfalfa biotypes and another three lines (P123, ArPo58, and S1PS02) identified as pea biotypes based on the plant from which they were collected and their genetic profiles at several polymorphic microsatellite loci (Peccoud et al., 2009). These aphid lines were collected in different locations and maintained in our lab on the universal host of *A. pisum*, *V. faba* (faba bean) (**Supplementary Table S1**). To confirm the assigned biotypes, we examined their fecundity on *M. sativa* (alfalfa), *P. sativum* (pea), and *V. faba*. Although there was variation in total nymph production between the lines, both biotypes produced a large number of nymphs on *V. faba* and their respective specific host plant but not on the nonspecific host plant (**Figure 1**). Hence, these six aphid lines showed distinct host specificity and served as a model system to examine biotype-specific gene expression patterns. We also showed that all the lines performed equally well on *V. faba* and their specific hosts, confirming the “universal host status” of *V. faba*.

### Candidate Salivary Effector Genes Were Identified From Two *A. Pisum* Lines

**Figure 2:**
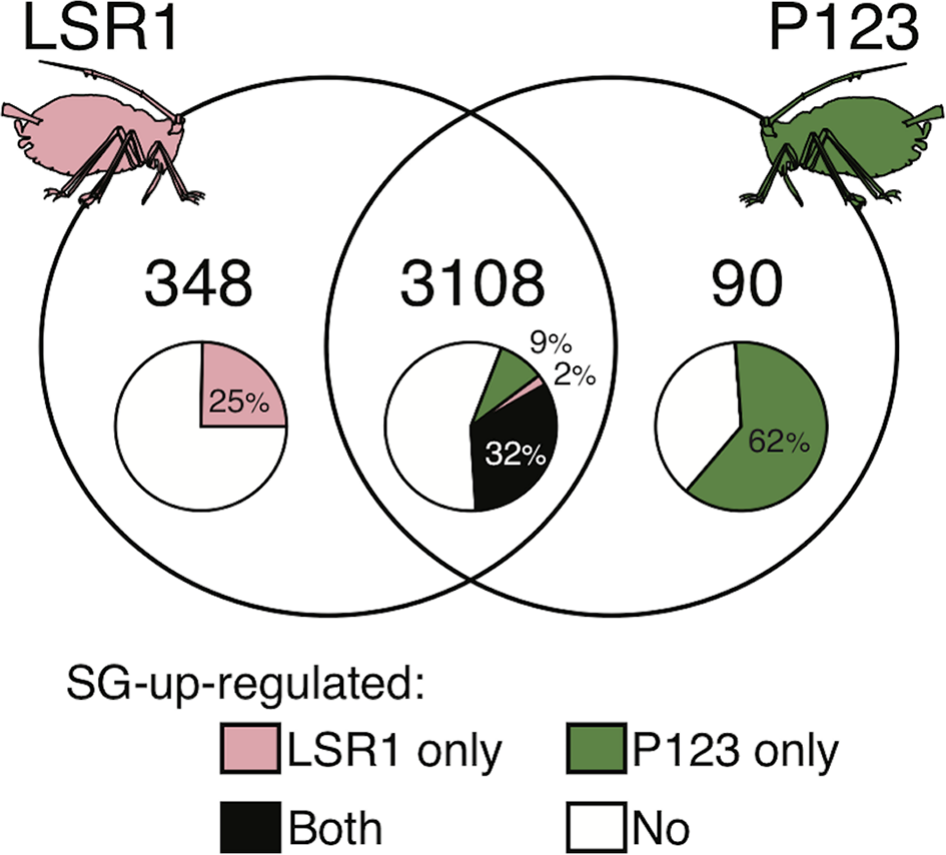
Candidate salivary effectors identified from salivary gland transcriptomes of LSR1 and P123 lines. Among the 3,546 candidate salivary effectors, 3,108 genes were expressed in both lines, 348 genes were expressed only in LSR1 and 90 genes were expressed only in P123. The pie charts indicate the proportions of salivary effectors that are upregulated in the salivary glands in comparison with the alimentary tract.

RNA-seq analysis of P123 (pea biotype) SG and AT samples along with LSR1 SG and AT samples (Boulain et al., 2018) retained 12,421 protein-coding genes for analysis and identified 3,546 genes that are expressed (CPM > 1) in SGs of at least one of the aphid lines and encoding proteins that are predicted to be secreted (Boulain et al., 2018). Out of the 3,546 candidate salivary effector genes, 3,108 genes were commonly expressed in SGs of the two aphid lines, while 348 and 90 genes were specifically expressed in LSR1 and P123, respectively (**Figure 2**). The comparison between the SG and AT samples from each aphid line allowed us to identify SG-upregulated genes among salivary effector genes. Among the 3,108 common salivary effectors, 32% (1,007 genes) were SG-upregulated in both LSR1 and P123 lines, whereas 2% (63 genes) and 9% (273 genes) were SG-upregulated only in LSR1 and P123, respectively. Out of the LSR1-specific salivary genes, 25% (86 genes) were upregulated in LSR1 SGs, whereas 62% (56 genes) of the P123-specific salivary effectors were upregulated in P123 SGs. The overlap between the two lines led to a total set of 1,485 SG-upregulated effector candidates (**Figure 2**, **Supplementary Table S3**).

There was a possibility of not detecting expression of P123-specific genes in this analysis because the LSR1 reference genome was used for mapping and counting the RNA-seq data. Therefore, we conducted *de novo* assembly of SG RNA samples for each aphid line and mapped them on the reference genomes (LSR1 and the obligate symbiont, *Buchnera aphidicola*). LSR1 SG RNA samples produced 565 unmapped contigs (mean length 454 bp, median 284 bp) while P123 SG RNA samples produced 566 unmapped contigs (mean length 453 bp, median 276 bp), out of which 108 showed high homology to unmapped *de novo* assembled LSR1 contigs. Unmapped contigs were BLASTed against NCBI nonredundant protein sequences (**Supplementary Table S4**). More than 360 contigs in each sample had no BLAST hit and more than 120 contigs of each sample showed similarity with hypothetical or uncharacterized proteins. Since these unmapped contigs from two aphid lines showed similar numbers, short length, and high rate of no BLAST hit, we concluded that use of the LSR1 reference genome for mapping and counting the SG RNA-seq data would not miss a large number of P123-specific salivary genes, if they exist, and continued to use the reference genome for further study.

### A Large Majority of Candidate Salivary Effector Genes Was Detected in Head Samples

We reasoned that examination of gene expression patterns in multiple aphid lines that belong to the same biotype would distinguish biotype-specific gene expression patterns from line-specific expression patterns. However, dissection of SGs is difficult and preparation of SG samples for six aphid lines was not realistic for us. Hence, we decided to use head samples, which are easier to prepare compared to SG samples, to examine the expression patterns of candidate salivary effector genes. We examined expression patterns of the 3,546 candidate salivary effector genes in the SG and head samples of LSR1 and P123 (reared on *V. faba*). In both sets of samples, gene expression levels in SGs and heads were well correlated (**Supplementary Figure S4**), and 3,165 (91.6%) and 3,107 (97.1%) of candidate salivary effector genes identified for each line were detected in head samples of LSR1 and P123, respectively. Hence, the aphid head samples provide approximate information on the expression levels of salivary genes and can be exploited to identify the candidate salivary genes that are expressed in a biotype-specific manner. Note that none of the reads mapped to the eight aphid viral genomes; thus, no aphid line seemed to be infected by the viruses.

### Aphid Line and Biotype, But Not Host Plants, Had a Marked Effect on the Expression of Candidate Salivary Effector Genes 

The six aphid lines were reared on either *V. faba* or on their specific host plant (*M. sativa* and *P. sativum*, respectively, for alfalfa and pea biotypes) for 9 days and RNA of heads was prepared and subjected to RNA-seq analysis. A distance-based clustering analysis of global expression patterns showed a strong effect of aphid lines and biotypes whereas the clustering was not influenced by host plant (**Supplementary Figure S3C**). We tested the effects of the three factors (line, biotype, and plant) and identified DE genes due to each factor (**Table 1**, **Supplementary Table S3**). Only six and 12 genes were DE depending on the host plants in the alfalfa biotype and the pea biotype respectively. Two genes were commonly downregulated in the two aphid biotypes feeding on *V. faba* compared to the specific plants (*M. sativa* or *P. sativum*) and encoded a linear gramicidin synthase subunit D and an unknown protein. Out of the 16 DE genes, four encoded candidate effectors and all of these were upregulated in the pea biotype when they were feeding on *V. faba* compared to *P. sativum*. These four candidate effector genes (predicted to encode an uncharacterized protein, a dnaJ homolog subfamily B member 11, a probable low-specificity L-threonine aldolase 2, and an endoplasmic reticulum resident protein 44), as well as the rest of the plant DE genes encoded seemingly unrelated proteins (**Supplementary Table S3**). Meanwhile 689 and 7,207 genes were DE depending on aphid biotype and line, respectively. More than one third (255) of the genes that showed biotype-specific differential expression patterns were candidate salivary effector genes (**Table 1**, **Supplementary Table S3**).

**Table 1:** Differentially expressed genes in the head samples of the alfalfa and pea biotypes reared on the universal and specific host plants.

Contrast	# of DE genes^a^	# of DE effector genes^b^
***Plant effect*** (universal vs. specific)		
• in alfalfa biotype	6	0
• in pea biotype Total	12 16	4 4
***Biotype effect*** (alfalfa vs. pea)^c^	689	255
***Line effect*** (line vs. line)^d^		
• in alfalfa biotype	6583	1934
• in pea biotype Total	2165 7207	697 2116

a Number of protein-coding genes that are differentially expressed with a FDR < 0.05 and a FC > 1.5 (from overall set of 18,601 protein coding genes existing in the NCBI Acyrthosiphon pisum Annotation Release 102).

b Number of differentially expressed genes that are considered as candidate salivary effectors (from overall set of 3,546 candidate salivary genes).

c In addition to FDR and FC filtering, a Student t-test was applied to exclude DE genes that showed high intra-biotype variability.

d The line effect is computed independently in each biotype and a gene is considered as DE when a FDR < 0.05 and a FC > 1.5 are observed between at least two of the three lines that constitute each biotype.

### Biotype-Specific DE Salivary Effector Sets Were Enriched With Duplicated and Aphid-Specific Genes 

**Figure 3:**
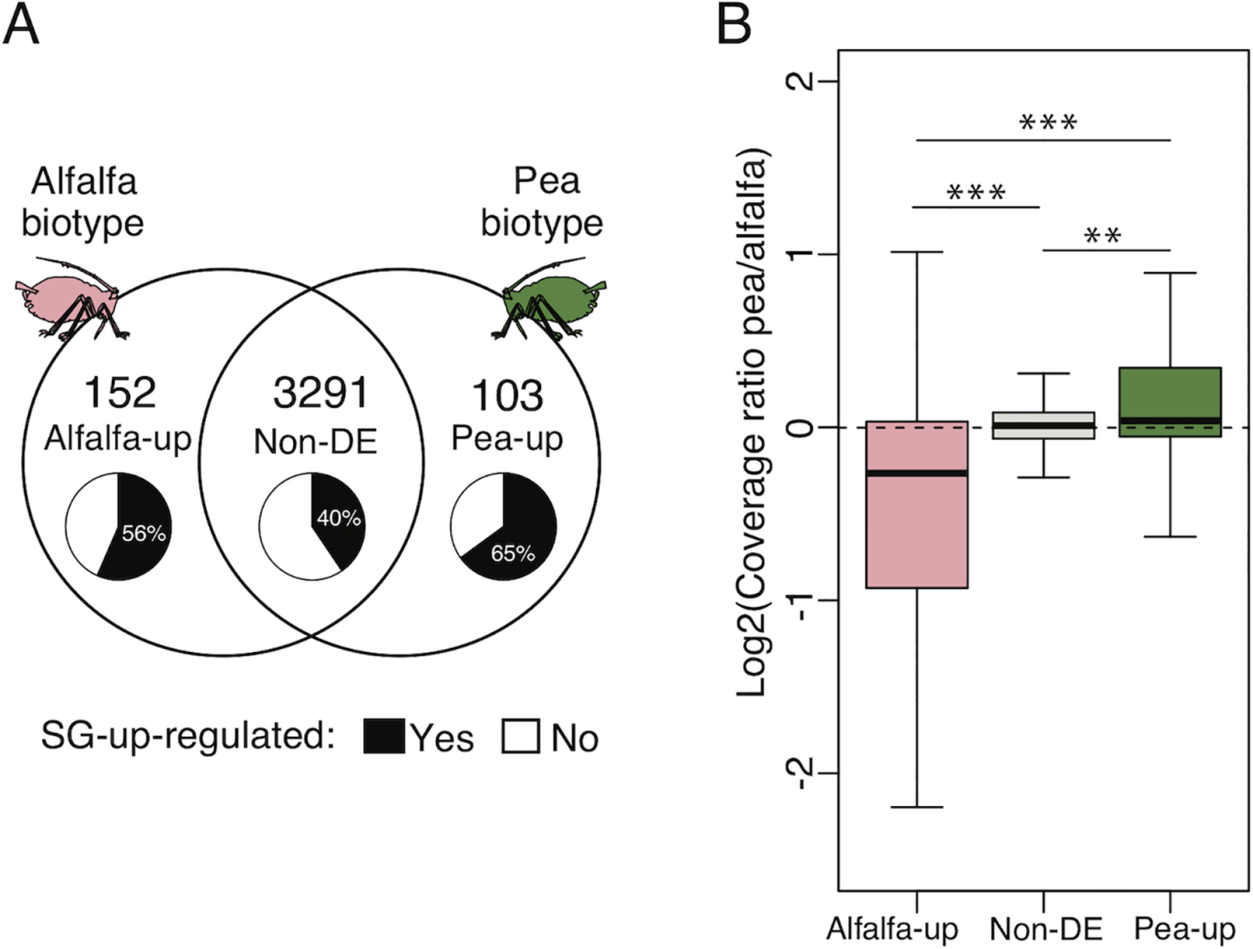
Candidate salivary effector genes in pea and alfalfa biotypes and their genome sequence coverage. **(A)** The 3,546 candidate salivary effector genes identified from salivary glands of LSR1 and P123 were categorized in three groups based on their DE patterns in the head samples of the alfalfa and pea biotypes. 152 salivary genes were upregulated in the alfalfa biotype, and 103 genes were upregulated in the pea biotype, while 3,291 genes were not differentially expressed. The pie charts indicate the proportions of salivary effector genes showing upregulation in SGs compared with ATs in at least one of the reference aphid lines, LSR1 or P123. **(B)** Genome sequence coverage ratio (pea/alfalfa) of salivary genes was determined by mapping of pool-seq reads on the LSR1 genome. Asterisks indicate statistical differences after Mann-Whitney tests between alfalfa-up, pea-up, and non-DE salivary effector subsets (**P < 0.01, ***P < 0.001).

Out of the 3,546 candidate salivary effector genes identified from the SGs of LSR1 and P123 reference lines, 152 were significantly upregulated in the alfalfa biotype (alfalfa-up) compared to the pea biotype, and 103 were upregulated in the pea biotype (pea-up) compared to the alfalfa biotype (**Figure 3A**). The rest of the 3,291 genes were not significantly DE between the two biotypes (non-DE). Among these alfalfa-up, pea-up, and non-DE subsets, 86 (56%), 67 (65%), and 1,332 (40%) candidate salivary effectors, respectively, were upregulated in the SGs compared to the ATs in at least one of the two reference lines.

**Figure 4:**
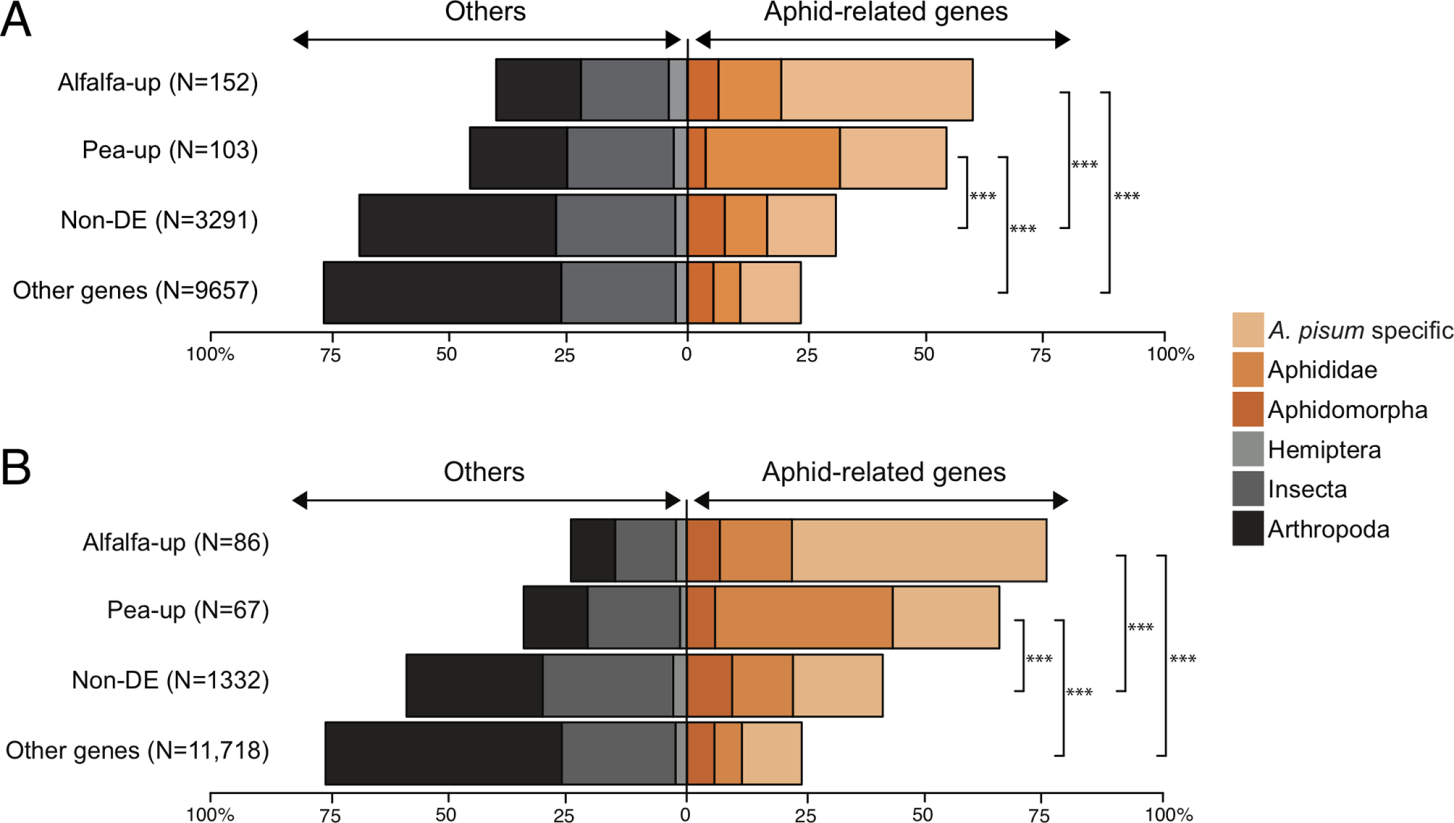
Orthology profiles of candidate salivary **(A)** and salivary upregulated **(B)** effector sets. Proportions of the different orthologous categories in the salivary effector sets and the other *A. pisum* genes that were considered as expressed in this study are shown. Asterisks indicate the significance of differences in the proportion of genes that belong to the same orthologous categories (proportion test, ***P < 0.001). Orthologous categories were assigned by Boulain et al. (2018), based on an OrthoDB analysis using 17 insect genomes.

Orthology analysis showed that both alfalfa-up and pea-up salivary effector sets contained high proportions of aphid lineage-specific genes compared to the non-DE sets and the other genes that were not considered as candidate salivary effector genes (**Figure 4A**). The proportion of aphid lineage-specific genes was even higher (>60%) in the alfalfa-up and pea-up subsets when only SG-upregulated genes of each category were considered (**Figure 4B**). The alfalfa-up and pea-up sets contained 79 (52%) and 57 (55%) genes that encode uncharacterized proteins, respectively (**Supplementary Table S3**).

In our previous study, we found that *A. pisum* candidate salivary effector genes contained multiple members of multigene families (Boulain et al., 2018). Thus, we examined whether the alfalfa-up or pea-up subsets contained more duplicated genes than expected by chance alone (tested on genes having at least one paralogue). The observed numbers of duplicated genes in the two subsets always lay above the 95% CI, reflecting a higher number of duplicated genes than expected (alfalfa-up: 65 genes, 95% CI = [32, 52] and pea-up: 42 genes, 95% CI = [20, 38]). In contrast, the non-DE subset contained fewer duplicated genes than expected as the number of observed genes lay below the 95% CI (894 genes, CI = [915, 942]).

**Figure 5:**
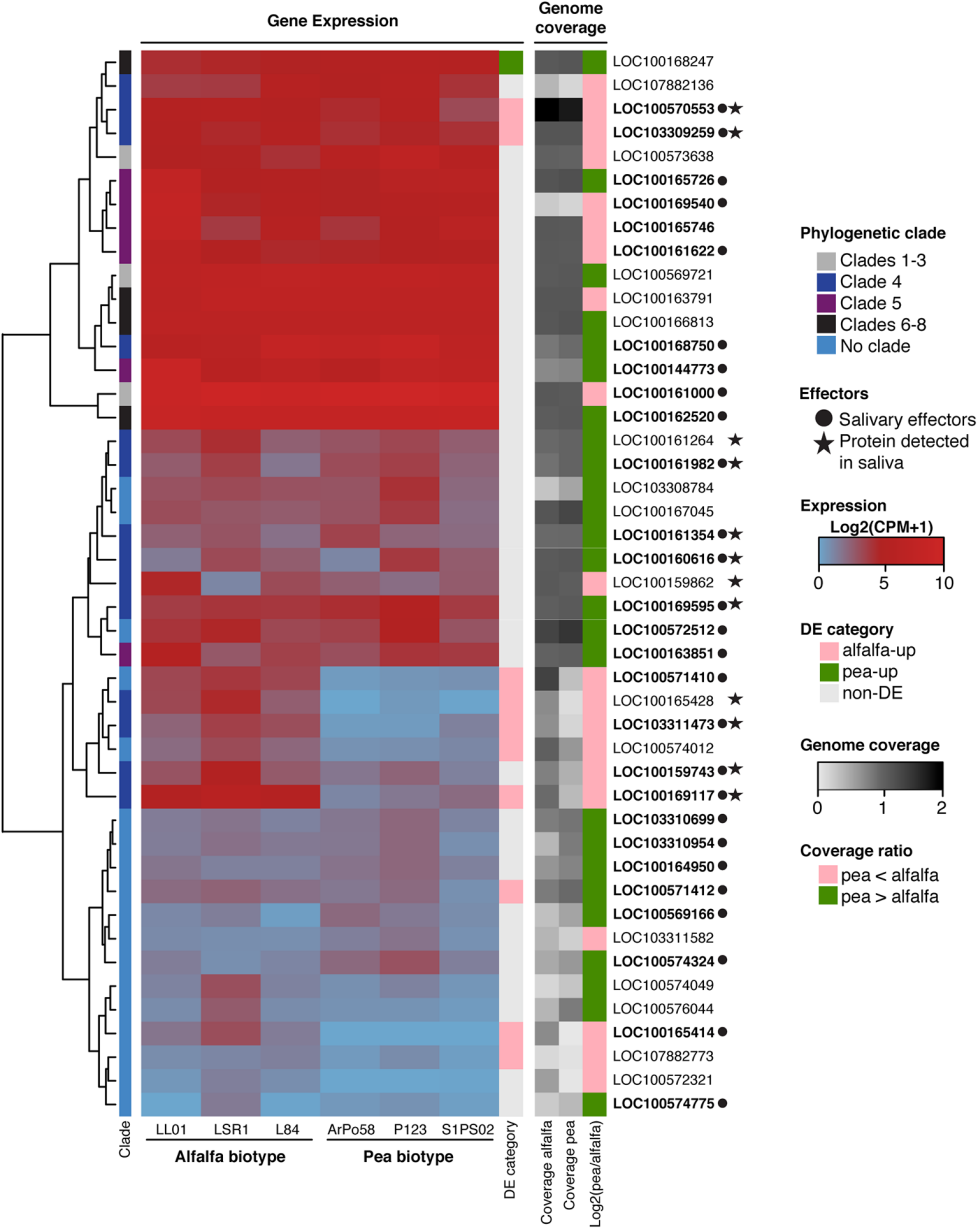
Gene expression and genome sequence coverage of Aminopeptidase-N gene family. The tree on the left is a hierarchical clustering of expression values and squares on the right side of the tree correspond to the clades of Aminopepdidase-N members assigned by Boulain et al. (2018). A heatmap of expression levels of Aminopeptidase-N family members in various aphid lines is shown in the middle. DE categories and sequencing coverage of the genomes and NCBI accession numbers are shown on the right. The genes were considered as candidate salivary effector genes if they were present in the 3,546 set established by the transcriptomes of LSR1 and P123 salivary glands. Detection of proteins in aphid saliva was reported by Boulain et al. (2018) and Carolan et al. (2009).

Among these duplicated genes, a subset of the *A. pisum*-expanded Aminopeptidase-N gene family showed a clear biotype-specific expression pattern (**Figure 5**). Out of the 27 Aminopeptidase-N proteins that are predicted to be effectors (Boulain et al., 2018), seven were included in the alfalfa-up set while the remaining 20 were included in the non-DE set. Moreover, these alfalfa-up Aminopeptidase-N genes were classified as either “clade 4”, in which episodic events of positive selection have been reported, or “no clade” due to their diversified sequences (Boulain et al., 2018). Many Aminopeptidase-N genes with no assigned clade were lowly expressed in both biotypes while more than half of the genes classified to other clades were highly expressed in both biotypes.

### Differential Expression of Candidate Salivary Genes Is Associated With Copy Number Variation Between the Two Biotypes

As the biotype-specific differential expression of salivary genes may result from copy number variation between the alfalfa and pea biotypes, we examined the sequence coverage of the genomes of the two biotypes using the genomic pool-seq data created previously (Nouhaud et al., 2018). Comparison of the sequencing coverage ratio between the two biotypes revealed copy number variation. Mean coverage ratio (pea/alfalfa) of the alfalfa-up set was significantly lower than the non-DE set and that of the pea-up set was significantly higher. This pattern was observed among the salivary effectors (**Figure 3B**) as well as in the SG-upregulated effectors (**Supplementary Figure S5**). The coverage of four alfalfa-up salivary effector genes was very low in the pool-seq of the pea biotype (<0.1), and some of these genes were very lowly expressed in the SG of P123 and the head samples of the three pea biotype lines (CPM < 1), while they were expressed (CPM > 1) in the three lines of the alfalfa biotype. These genes were predicted to encode an Aminopeptidase-N-like protein, a fatty acid synthase-like protein, a ubiquitin-C-like protein, and an uncharacterized protein. Those genes may not be encoded in the genome of the pea biotype lines and be specific to the alfalfa biotype although their predicted functions do not seem to be related. No such gene (very low coverage and expression value in the alfalfa biotype) was observed in the pea-up gene set (**Supplementary Table S3**).

## Discussion

To understand the molecular basis of host plant adaptation in *A. pisum* biotypes, we created a comprehensive list of candidate salivary genes using two aphid lines that belong to the pea or alfalfa biotype and compared their expression patterns in the two biotypes, each represented by three genetically distinct aphid lines. Due to the difficulty of creating SG RNA samples, we used aphid head samples to examine biotype-specific expression patterns of candidate salivary genes and the effect of host plants. Comparison of gene expression levels in the head and SG samples showed that expression levels of the majority of genes were correlated between the two sample types with some exceptions. The head samples contain many organs (eyes, antennae, brain, etc.) in addition to SGs. Some of the SG-expressed genes might be expressed in other organs than the SGs and, in such cases, correlation between the expression values in the SGs and the heads is not expected. Nonetheless, this study presents one of the most thorough and comparative analyses of salivary gene expression in genetically related insect lines with clearly distinct host plant specificity.

The analyses of the head samples showed strong line and biotype effects on aphid gene expression and revealed a very weak effect of host plant. Our results are in line with the study of Eyres et al. (2016), which examined transcriptional patterns of six pea aphid biotypes reared on their specific and universal host plants and found little expression change caused by host plant type. Unlike the generalist aphid *M. persicae*, which shows large changes in gene expression to acclimatize to host plant (Mathers et al., 2017), *A. pisum* biotypes seem to make very little transcriptional adjustment to their host plants. This difference in transcriptional plasticity may explain the differences in host range of the two aphid species (generalist vs. specialist) although further examination of multiple generalist and specialist aphids are required to link the transcriptional plasticity with host range.

We focused our analyses on the expression patterns of the candidate salivary gene sets created by LSR1 and P123 SG transcriptomes. Although the effect of aphid line on gene expression patterns was considerable, we were able to identify 153 and 103 candidate salivary genes that are upregulated in the alfalfa and the pea biotypes, respectively. Differential expression of salivary genes in six *A. pisum* biotypes was reported previously using a smaller list of candidate salivary genes (307 genes) published at the time and by using multiple aphid lines as biological replicates of a biotype (Eyres et al., 2016). Our study refined the analysis by creating and compiling biotype-specific salivary gene sets for an alfalfa- and a pea-adapted *A. pisum* line, by expanding the candidate salivary genes list by more than 10 times and by including three biological replicates for each aphid line and for each condition.

The orthology analysis of candidate salivary genes revealed that the alfalfa-up and pea-up gene sets contain higher proportions of aphid lineage-specific genes and the proportion of those genes was even higher when only the SG-upregulated salivary genes were analysed. These alfalfa-up and pea-up gene sets also contain higher numbers of duplicated genes than expected. These observations support the scenario that biotype-specific salivary effectors may have evolved recently and diversified through duplication events, possibly in relation to the diversification of the pea aphid complex of biotypes (Peccoud et al., 2009). Under this scenario, certain gene duplicates would tend to be recruited differently in the pea and alfalfa biotypes to achieve better performance on each host plant while other copies would maintain basic functions and lie in the non-DE set. Analysis of the gene family of Aminopeptidase-N supports this scenario as it revealed a subset of genes that show high expression values in all the six aphid lines and another set of genes that show differential expression in a biotype specific manner.

Four alfalfa-up salivary genes showed virtually no expression values in both heads and SGs and low genome coverage in the pea biotype. These genes may not exist in the three lines of pea biotype studied here and be considered as alfalfa biotype-specific genes. Although the expression levels of those genes in the alfalfa biotype tend to be low, they may be required for efficient feeding on alfalfa plants or may trigger unwanted responses in pea plants. On the contrary, all the pea-up genes were highly expressed in the pea biotype and all of them seem to be encoded in the alfalfa biotype genome. Our analysis of *de novo* assembled transcripts showed very little difference between LSR1 and P123 lines. Although there is a possibility that we missed some genes that are specifically encoded in the pea biotype and absent in the alfalfa biotype by using the LSR1 genome as reference for the RNA-seq analyses, the number of such genes should be very small. Thus, except for a few biotype-specific genes, the repertoires of the salivary genes in the two biotypes were almost identical, and small sets of genes showed differential expression which might determine host plant specificity. The evolutionary history of specialization to *P. sativum* and *M. sativa* in *A. pisum* biotypes has not been elucidated, and we cannot speculate on the evolutionary process of the differential expression (gene loss vs. gain, induction vs. suppression) of these genes.

Although the effect of biotype on salivary effector expression was small, the host plants showed an even smaller effect on effector transcription. This suggests that the gene expression differences in candidate salivary effectors between the biotypes largely result from genomic variation and not from expression plasticity. This is supported by another result showing a low ratio of genome coverage (pea/alfalfa) for the alfalfa-up gene set and a higher ratio for the pea-up gene set: differential expression of these two sets of genes can be partly explained by copy number variation in the two biotypes. In addition to copy number variation, variation of coding sequences or promoter regions (small insertion/deletion/inversion, SNPs) and gene rearrangements may also be the causes of differential expression of candidate salivary effectors between the biotypes. As genome sequences of different aphid lineages and a better assembly of the pea aphid genome are becoming available (Nouhaud et al., 2018; Li et al., 2019), dedicated studies are needed for a thorough investigation of biotype-specific amino acid sequence polymorphism of candidate effectors and potential causes of differential gene expression.

In conclusion, this study provides a comprehensive list of candidate salivary effectors and brings evidence that a subset of salivary genes that include a high proportion of aphid lineage-specific genes and duplicated genes are DE in two aphid biotypes with distinct host specificity. The identified DE salivary genes are strong candidate genes that might be involved in host plant adaptation in the *A. pisum* biotypes and deserve further functional characterization.

## Data Availability Statement

The datasets generated and analysed for this study can be found in the NCBI using accession numbers: SRX3969578, SRX3969577, SRX3969576, SRX5933263, SRX5933264, SRX5933265, SRX3969582, SRX3969581, SRX3969580, SRX5933266, SRX5933267, SRX5933268, SRX5936232, SRX5936233, SRX5936234, SRX5936235, SRX5936236, SRX5936237, SRX5936238, SRX5936239, SRX5936240, SRX5936240, SRX5936248, SRX5936249, SRX5936246, SRX5936247, SRX5936252, SRX5936253, SRX5936250, SRX5936251, SRX5936244, SRX5936245, SRX5936228, SRX5936227, SRX5936226, SRX5936225, SRX5936224, SRX5936223, SRX5936222, SRX5936221, SRX5936230, SRX5936229, SRX5936242, SRX5936243, SRX5936231.

## Author Contributions

EG, SM, J-CS, and AS designed the experiments. HB, FL, JJ, EG, SM, J-CS, and AS conducted the experiments, analyzed the data, and wrote the manuscript. J-CS and AS provided funding for the project.

## Funding

This work was funded by the Agence Nationale de la Recherche (ANR) Bugspit (ANR-13-JSV7-0012-01) to AS and by the ANR Speciaphid (ANR-11-BSV7-005-01) to J-CS.

## Conflict of Interest

The authors declare that the research was conducted in the absence of any commercial or financial relationships that could be construed as a potential conflict of interest.
