# Supplementary material for: Differential Expression of Candidate Salivary Effector Genes in Pea Aphid Biotypes With Distinct Host Plant Specificity
Source: Front Plant Sci. 2019 Oct 22;10:1301. doi: 10.3389/fpls.2019.01301 (PMC6818229; doi:10.3389/fpls.2019.01301)
Supplement: Supplementary file 1 [file DataSheet_1.zip › Data Sheet 1.PDF]

TABLE S1 | Lines of *A. pisum* used in this study.

Line	Color	Plant of collection	Location	Collection date	Secondary symbiont	References
ArPo58	Green	Pea	Ardillières, France	November 2011	none	Guy et al., 2016
P123*	Green	Pea	Mauze-le-Mignon, France	April 1999	none	Simon et al., 2011
S1PS02	Green	Pea	St Prex, Switzerland	May 2012	none	this study
LSR1	Pink	Alfalfa	Ithaca, USA	October 2007	none	IAGC, 2010
LL01	Green	Alfalfa	Lusignan, France	January 1987	none	Sabater-Muñoz., 2006
L84*	Green	Alfalfa	Lusignan, France	March 1999	none	this study

* treated by ampicillin in 2010 and secondary symbionts were removed

TABLE S2 | NCBI accession numbers of RNA-seq libraries generated in this study.

Sample name	Dissected tissues	Aphid line	Rearing plant	Number of reads[a]	Number of mapped reads[b]	Mapping rate (%)	SRA accession
SG_LSR1	Salivary glands	LSR1	*Vicia faba*	31002452	21775316	70,2	SRX3969578
SG_LSR1	Salivary glands	LSR1	*Vicia faba*	33174622	24290996	73,2	SRX3969577
SG_LSR1	Salivary glands	LSR1	*Vicia faba*	32257454	24655372	76,4	SRX3969576
SG_P123	Salivary glands	P123	*Vicia faba*	32508352	23536798	72,4	SRX5933263
SG_P123	Salivary glands	P123	*Vicia faba*	29972192	20461966	68,3	SRX5933264
SG_P123	Salivary glands	P123	*Vicia faba*	39377298	28991870	73,6	SRX5933265
AT_LSR1	Alimentary tract	LSR1	*Vicia faba*	28637176	22125482	77,3	SRX3969582
AT_LSR1	Alimentary tract	LSR1	*Vicia faba*	32941902	27059246	82,1	SRX3969581
AT_LSR1	Alimentary tract	LSR1	*Vicia faba*	34419002	29087704	84,5	SRX3969580
AT_P123	Alimentary tract	P123	*Vicia faba*	31320696	24704742	78,9	SRX5933266
AT_P123	Alimentary tract	P123	*Vicia faba*	32778284	25573806	78,0	SRX5933267
AT_P123	Alimentary tract	P123	*Vicia faba*	33434694	27481658	82,2	SRX5933268
LSR1_Vf	Head	LSR1	*Vicia faba*	38886078	31592740	81,2	SRX5936232
LSR1_Vf	Head	LSR1	*Vicia faba*	35741992	29409480	82,3	SRX5936233
LSR1_Vf	Head	LSR1	*Vicia faba*	38063296	31582710	83,0	SRX5936234
LSR1_Ms	Head	LSR1	*Medicaco sativa*	31302318	24553436	78,4	SRX5936235
LSR1_Ms	Head	LSR1	*Medicaco sativa*	32487740	26236628	80,8	SRX5936236
LSR1_Ms	Head	LSR1	*Medicaco sativa*	34354212	26634640	77,5	SRX5936237
LL01_Vf	Head	LL01	*Vicia faba*	35500594	25862100	72,8	SRX5936238
LL01_Vf	Head	LL01	*Vicia faba*	33523786	18284594	54,5	SRX5936239
LL01_Vf	Head	LL01	*Vicia faba*	35565282	15461060	43,5	SRX5936240
LL01_Ms	Head	LL01	*Medicaco sativa*	32574234	23868042	73,3	SRX5936240
LL01_Ms	Head	LL01	*Medicaco sativa*	31190910	20029482	64,2	SRX5936248
LL01_Ms	Head	LL01	*Medicaco sativa*	34328558	20018826	58,3	SRX5936249
L84_Vf	Head	L84	*Vicia faba*	35329908	27588582	78,1	SRX5936246
L84_Vf	Head	L84	*Vicia faba*	29975554	22426852	74,8	SRX5936247
L84_Vf	Head	L84	*Vicia faba*	36237742	27655528	76,3	SRX5936252
L84_Ms	Head	L84	*Medicaco sativa*	33076804	24341828	73,6	SRX5936253
L84_Ms	Head	L84	*Medicaco sativa*	32058060	21783302	67,9	SRX5936250
L84_Ms	Head	L84	*Medicaco sativa*	40563632	30364542	74,9	SRX5936251
ArPo58_Vf	Head	ArPo58	*Vicia faba*	31237870	20013444	64,1	SRX5936244
ArPo58_Vf	Head	ArPo58	*Vicia faba*	29674748	18885532	63,6	SRX5936245
ArPo58_Vf	Head	ArPo58	*Vicia faba*	38804080	26434428	68,1	SRX5936228
ArPo58_Ps	Head	ArPo58	*Pisum sativum*	30486306	19942130	65,4	SRX5936227
ArPo58_Ps	Head	ArPo58	*Pisum sativum*	32923208	23035770	70,0	SRX5936226
ArPo58_Ps	Head	ArPo58	*Pisum sativum*	36825156	25870098	70,3	SRX5936225
P123_Vf	Head	P123	*Vicia faba*	33236354	24828562	74,7	SRX5936224
P123_Vf	Head	P123	*Vicia faba*	32945154	24732590	75,1	SRX5936223
P123_Vf	Head	P123	*Vicia faba*	32917792	25554808	77,6	SRX5936222
P123_Ps	Head	P123	*Pisum sativum*	33479638	28305054	84,5	not submitted*
P123_Ps	Head	P123	*Pisum sativum*	36831628	31318418	85,0	not submitted*
P123_Ps	Head	P123	*Pisum sativum*	37084682	31214236	84,2	SRX5936221
S1PS02_Vf	Head	S1PS02	*Vicia faba*	36782652	29296658	79,6	SRX5936230
S1PS02_Vf	Head	S1PS02	*Vicia faba*	33775718	26892466	79,6	SRX5936229
S1PS02_Vf	Head	S1PS02	*Vicia faba*	33031406	27185234	82,3	SRX5936242
S1PS02_Ps	Head	S1PS02	*Pisum sativum*	28573866	19534478	68,4	SRX5936243
S1PS02_Ps	Head	S1PS02	*Pisum sativum*	31675596	26562542	83,9	not submitted*
S1PS02_Ps	Head	S1PS02	*Pisum sativum*	29055808	19576112	67,4	SRX5936231

*These three libraries were not submitted to NCBI and not used in this study as clustering analysis revealed that they were too divergent from the other replicates of the same condition

[a]Total number of reads (only pairs with both reads passing quality filtering were kept)

[b]Filtered reads were mapped on the Acyr_2.0 reference genome assembly of *Acyrthosiphon pisum* (GCF_000142985.2)

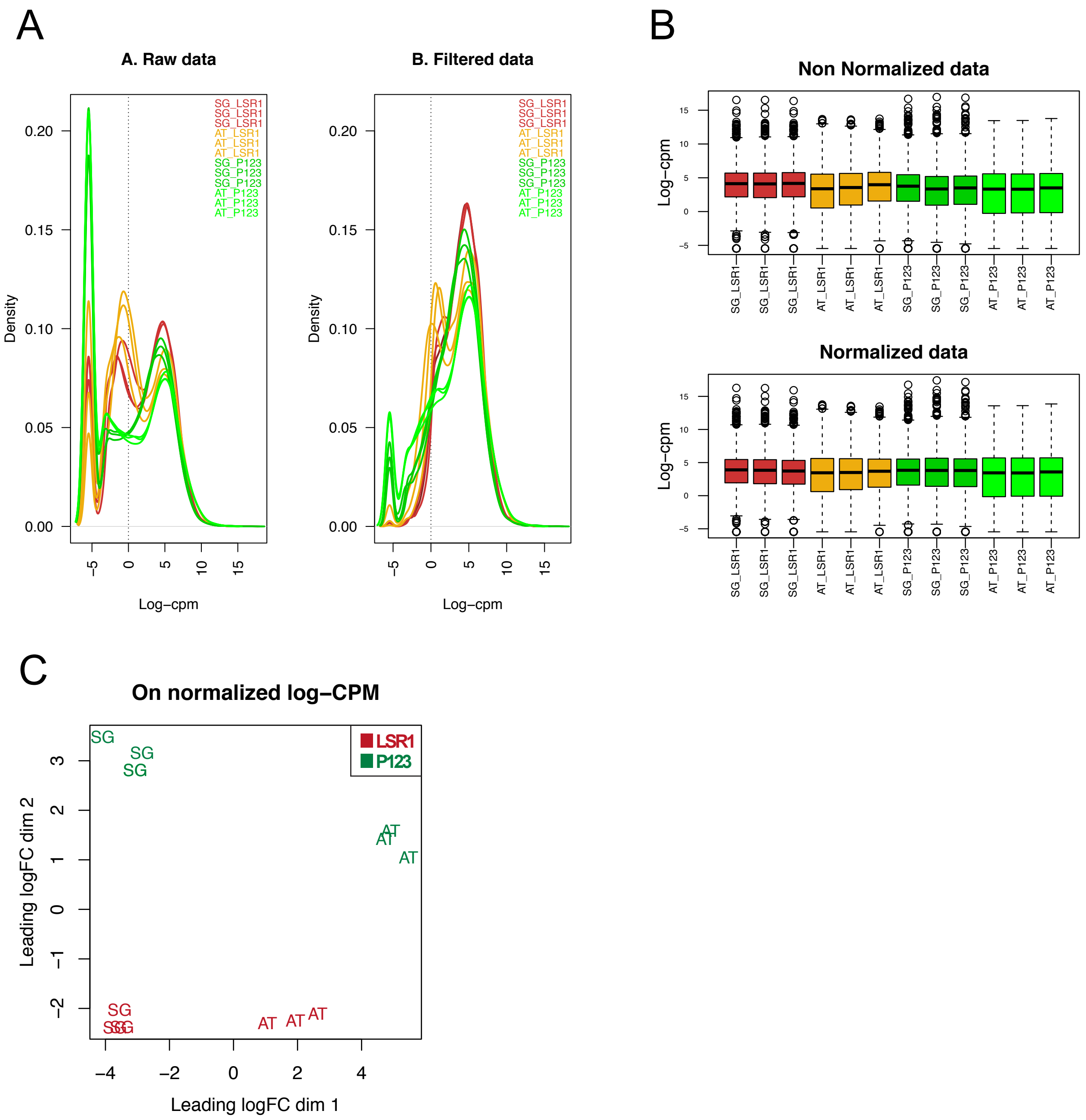

FIGURE S1 | Gene expression analysis of SGs and ATs from *A. pisum* LSR1 and P123 lines. (A) Density plot representing expression levels in log-cpm of all expressed genes (Raw data) and after removing the genes that do not meet the criteria of CPM>1 in at least 3 libraries of the12 analyzed libraries (Filtered data). **(B)** Expression level distribution of the expressed genes (filtered) before and after CPM normalization using TMM method for Normalization Factor calculation. After normalization, the distribution of gene expression of the 12 samples was similar. **(C)** Reproducibility of biological replicates. Multidimensional Scaling (MDS) plot of log-cpm over dimensions 1 and 2. The unsupervised clustering of sample groups did not show the presence of outliers, then all samples were retained.

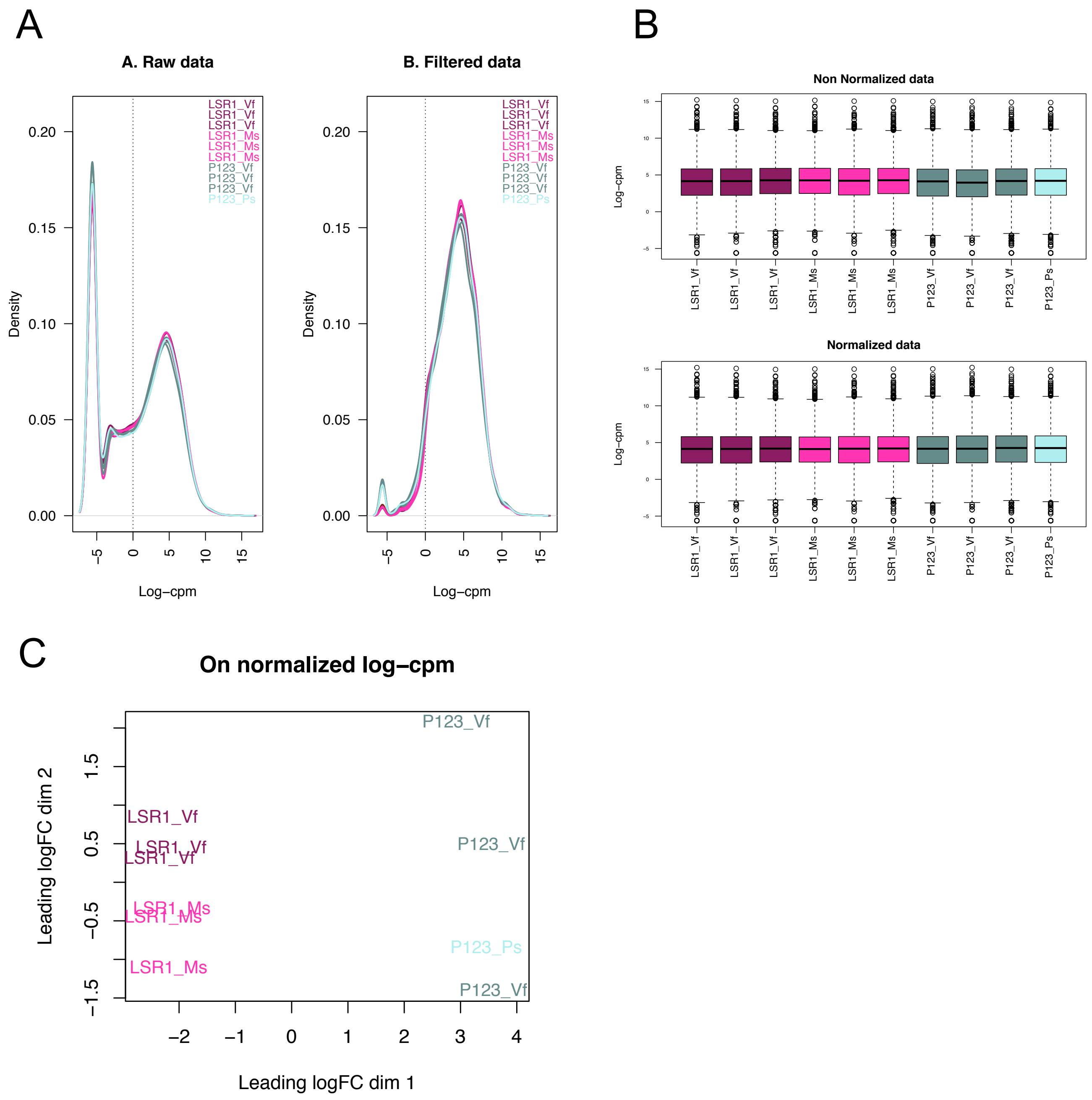

FIGURE S2 | Gene expression analysis of heads from *A. pisum* LSR1 and P123 lines feeding on the universal host *V. faba* (Vf) and their respective specific hosts, *M. sativa* (Ms) and *P. sativum* (Ps). Two head samples from P123 line on *P. sativm* were not clustering together and were removed from the final analysis. **(A)** Density plot representing expression levels in log-cpm of all expressed genes (Raw data) and after removing the genes that do not meet the criteria of CPM>1 in at least 3 libraries of the 10 analyzed libraries (Filtered data). **(B)** Expression level distribution of the expressed genes (filtered) before and after CPM normalization using TMM method for Normalization Factor calculation. After normalization, the distribution of gene expression of the 10 samples was similar. **(C)** Reproducibility of biological replicates. Multidimensional Scaling (MDS) plot of log-cpm over dimensions 1 and 2. The unsupervised clustering of sample groups did not show the presence of outliers, then all samples were retained.

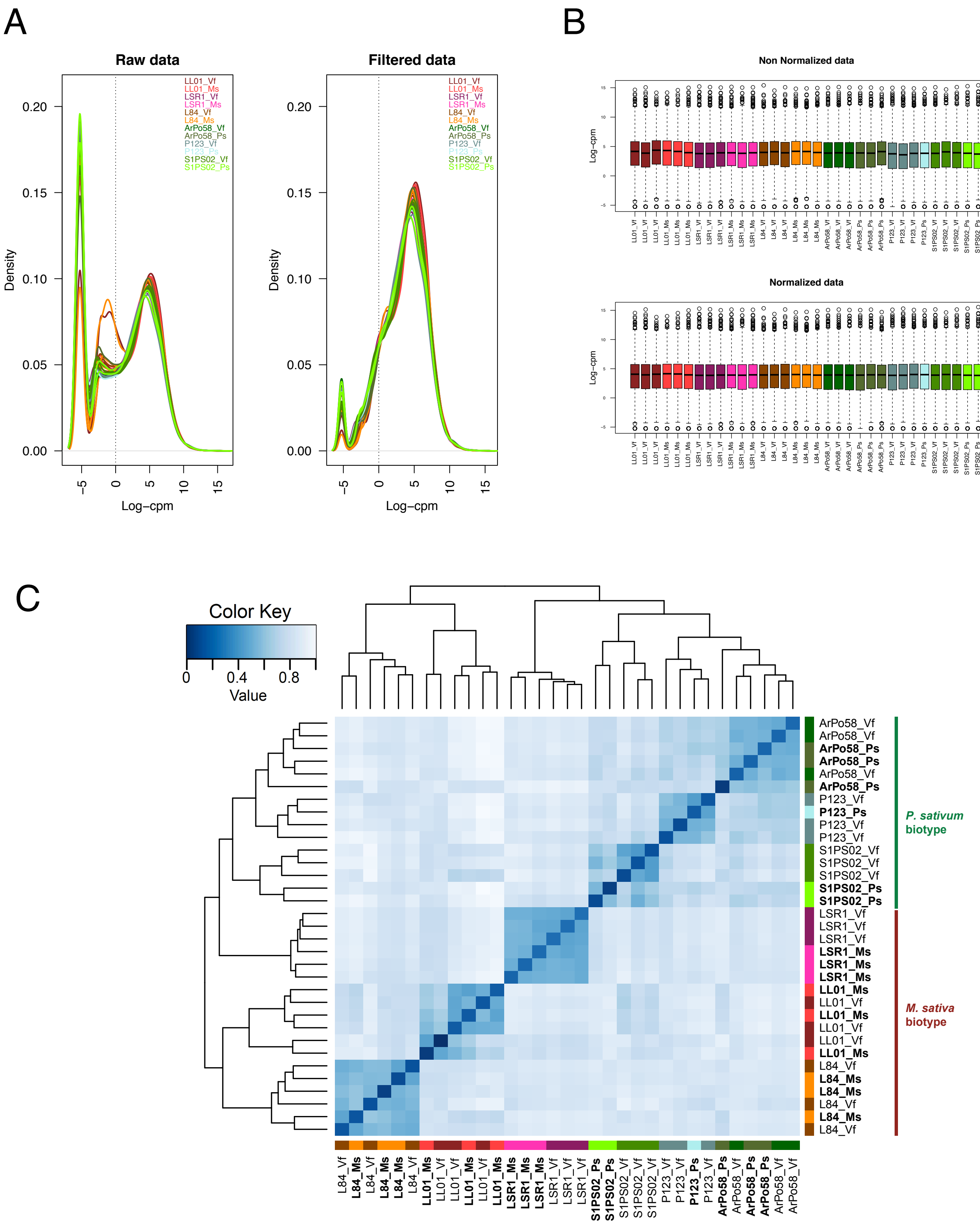

FIGURE S3 | Gene expression analysis of the 33 heads samples from the six lines of *A. pisum* belonging to two biotypes and feeding on the universal host *V. faba* (Vf) and their respective specific hosts, *M. sativa* (Ms) and *P. sativum* (Ps). Three samples (two P123_Ps samples and one S1PS02_Ps) were not clustering together and were removed from the final analysis. **(A)** Density plot representing expression levels in log-cpm of all expressed genes (Raw data) and after removing the genes that do not meet the criteria of CPM>1 in at least 3 libraries on the 33 analyzed libraries (Filtered data). **(B)** Expression level distribution of the expressed genes (filtered) before and after CPM normalization using TMM method for Normalization Factor calculation. After normalization, the distribution of gene expression of the 33 samples was similar. **(C)** Reproducibility of biological replicates. Heatmap of distances based on normalized CPMs. The clustering of sample groups did not show the presence of outliers.

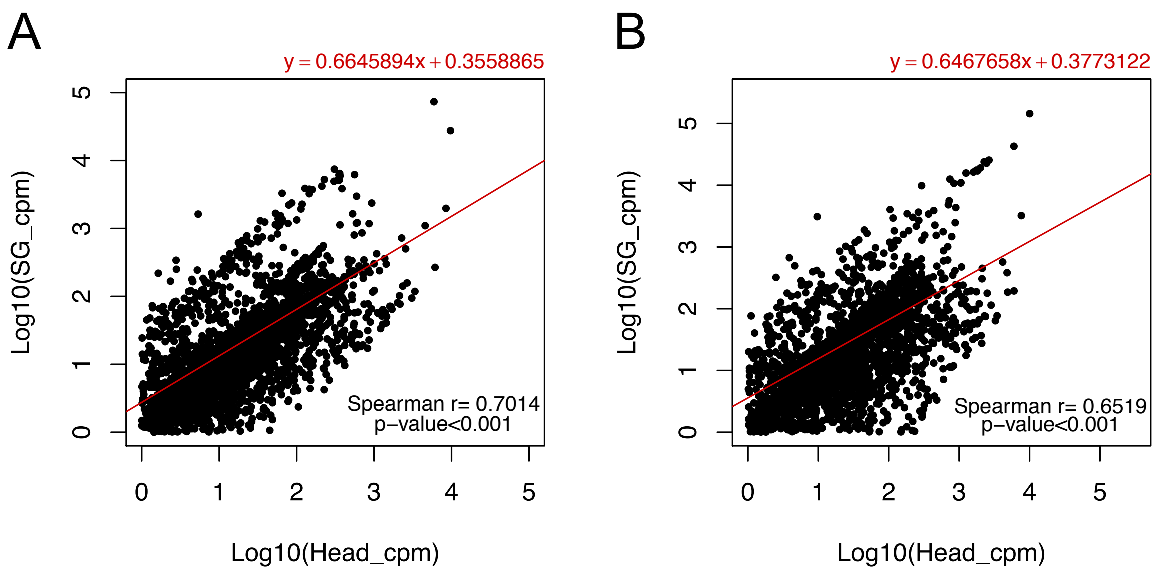

FIGURE S4 | Correlation between gene expression in salivary glands and heads for LSR1 (A) and P123 (B) salivary effector candidates. Only 3165 and 3107 salivary effector genes that were detectable in heads (CPM>1) of LSR1 and P123, respectively, were considered. Correlation coefficients were calculated with Spearman's method after the assessment of the distributions non-normality by Shapiro-Wilk normality test and QQ-plot viewing.

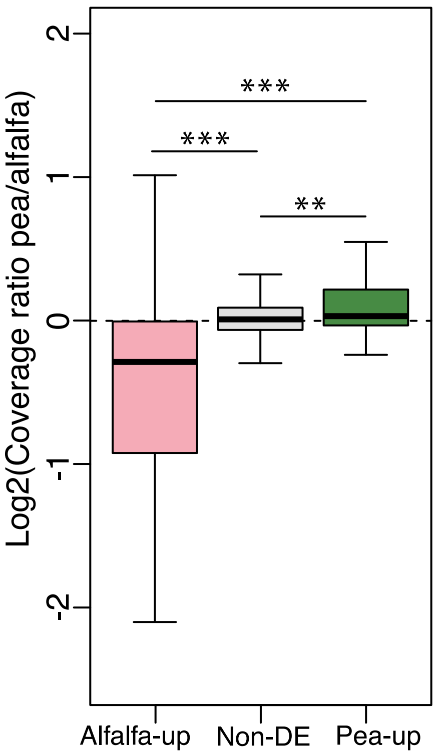

FIGURE S5 | Genome sequence coverage ratio of salivary up-regulated effectors in pea and alfalfa biotypes. Genome sequence coverage ratio (coverage in pea biotype/alfalfa biotype) of the three DE subsets of salivary up-regulated effector genes in alfalfa and pea biotypes (the 1485 candidate salivary up-regulated effector genes identified from salivary glands of LSR1 and P123 were considered). Genome coverage was determined by mapping of pool-seq reads on LSR1 genome. Asterisks indicate statistical differences after Mann-Whitney tests between alfalfa-up, pea-up and non-DE salivary effector subsets (**: $p<0.01$, ***: $p<0.001$).

References

Guy, E., Boulain, H., Aigu, Y., Le Pennec, C., Chawki, K., Morlière, S., et al. (2016). Optimization of agroinfiltration in *Pisum sativum* provides a new tool for studying the salivary protein functions in the pea aphid complex. *Front. Plant Sci.* 7. doi:10.3389/fpls.2016.01171.

Sabater-Muñoz, B., Legeai, F., Rispe, C., Bonhomme, J., Dearden, P., Dossat, C., et al. (2006). Large-scale gene discovery in the pea aphid *Acyrthosiphon pisum* (Hemiptera). *Genome biol.* 7, R21. doi.org/10.1186/gb-2006-7-3-r21

Simon, J.-C., Boutin, S., Tsuchida, T., Koga, R., Le Gallic, J.-F., Frantz, A., et al. (2011). Facultative symbiont infections affect aphid reproduction. *PLOS ONE* 6, e21831. doi.org/10.1371/journal.pone.0021831

The International Aphid Genomics Consortium (2010). Genome sequence of the pea aphid *Acyrthosiphon pisum*. *PLOS biol.* 8, e1000313. doi.org/10.1371/journal.pbio.1000313
